# Performance Analysis of IoT-Based Sensor, Big Data Processing, and Machine Learning Model for Real-Time Monitoring System in Automotive Manufacturing

**DOI:** 10.3390/s18092946

**Published:** 2018-09-04

**Authors:** Muhammad Syafrudin, Ganjar Alfian, Norma Latif Fitriyani, Jongtae Rhee

**Affiliations:** 1 Department of Industrial and Systems Engineering, Dongguk University, Seoul 100-715, Korea; udin@dongguk.edu (M.S.); norma@dongguk.edu (N.L.F.); 2 u-SCM Research Center, Nano Information Technology Academy, Dongguk University, Seoul 100-715, Korea

**Keywords:** monitoring system, IoT-based sensor, big data processing, fault detection, DBSCAN, Random Forest

## Abstract

With the increase in the amount of data captured during the manufacturing process, monitoring systems are becoming important factors in decision making for management. Current technologies such as Internet of Things (IoT)-based sensors can be considered a solution to provide efficient monitoring of the manufacturing process. In this study, a real-time monitoring system that utilizes IoT-based sensors, big data processing, and a hybrid prediction model is proposed. Firstly, an IoT-based sensor that collects temperature, humidity, accelerometer, and gyroscope data was developed. The characteristics of IoT-generated sensor data from the manufacturing process are: real-time, large amounts, and unstructured type. The proposed big data processing platform utilizes Apache Kafka as a message queue, Apache Storm as a real-time processing engine and MongoDB to store the sensor data from the manufacturing process. Secondly, for the proposed hybrid prediction model, Density-Based Spatial Clustering of Applications with Noise (DBSCAN)-based outlier detection and Random Forest classification were used to remove outlier sensor data and provide fault detection during the manufacturing process, respectively. The proposed model was evaluated and tested at an automotive manufacturing assembly line in Korea. The results showed that IoT-based sensors and the proposed big data processing system are sufficiently efficient to monitor the manufacturing process. Furthermore, the proposed hybrid prediction model has better fault prediction accuracy than other models given the sensor data as input. The proposed system is expected to support management by improving decision-making and will help prevent unexpected losses caused by faults during the manufacturing process.

## 1. Introduction

Manufacturing plays an important role in economic development and is still considered crucial to economic growth in the globalization era [1,2]. It has a positive impact on the growth of both developed and developing countries [3,4]. Emerging technologies are utilized by the manufacturing industry to enhance the economic competitiveness of individual manufacturers and the sustainability of the entire industrial sector. The adoption of information and communication technology (ICT) in manufacturing enables a transition from traditional to advanced manufacturing processes [5]. Monitoring systems, as part of ICT application, play an important part in manufacturing process control and management. Recent developments in information technology enable the integration of various monitoring applications into one complex system for the whole supply chain [6]. In general cases, the application of a monitoring system plays an important role in predicting disease [7], improving production, reducing cost [8], and providing an early warning system [9,10]. Recent technologies such as Internet of Things (IoT)-based sensors can be utilized and integrated with monitoring systems. Studies have been conducted in the manufacturing industry and showed significant benefits from the use of IoT-based sensors for monitoring such as working condition improvements [11], error design prevention [12], fault diagnosis [13], quality prediction [14], and helping managers with better decision making [15].

With the increasing number of IoT sensing devices available, data generated from the manufacturing industry (i.e., process logs, events, images and sensor data) are expected to grow exponentially. This type of data is called “big data” [16]. Big data analysis has led to significant improvements in the manufacturing industry, such as reducing energy consumption [17], improving production scheduling and logistics planning [18], mitigating social risks [19], and facilitating better decision making [20]. Previous studies have shown significant benefits from several big data technologies in processing and storing large volumes of data quickly, such as with the application of Apache Kafka [21,22,23,24,25,26], Apache Storm [27,28,29,30,31], and NoSQL MongoDB [32,33,34,35,36,37]. Previous studies showed significant advantages from the integration of big data technologies such as reducing the processing time for home automation systems [38], providing effective and efficient solutions for processing IoT-generated data for smart cities [39], and handling large amounts of smart environmental data in real-time [40]. The aforementioned big data technologies have been integrated in data processing systems, resulting in significant advantages due to processing large amounts of streaming spatiotemporal data [41] as well as processing massive amounts of manufacturing sensor data efficiently [42]. Therefore, it is necessary to integrate Apache Kafka, Apache Storm, and MongoDB in big data processing systems for the manufacturing industry so that large amounts of streaming manufacturing sensor data can be promptly processed, stored, and presented in real-time.

Data (e.g., sensor data from the production line, environmental data, etc.) generated by the manufacturing industry needs to be analyzed to help managers with decision making. Machine learning methods can be considered advanced technology with great potential for data analysis and has been successfully applied in various areas such as fault detection [43], quality prediction [14,44], defect classification [45], and visual inspection [46]. In the case of fault prediction, machine learning algorithms such as Random Forest are highly effective in detecting abnormal events in a process and thus can help avoid productivity loss [47,48,49]. However, machine learning algorithms encounter problems with outlier data, which can reduce the accuracy of the classification model. Outlier detection can be applied to identify and remove outliers; thus, improving the performance of classification models [50,51]. One of the techniques used for outlier detection is Density-Based Spatial Clustering of Applications with Noise (DBSCAN) [52]. DBSCAN has been implemented in different fields and has been demonstrated to be effective at detecting true outliers [53,54]. The integration of DBSCAN-based outlier detection and Random Forest is necessary for more accurate detection of abnormal events during the manufacturing process.

The results of aforementioned studies have shown significant advantages of IoT-based sensor, big data technology, and machine learning models on improving decision-making for management. Nevertheless, there is no study on integration of the IoT-based sensor, big data technology, and machine learning models into a complete monitoring system specifically for automotive manufacturing. Thus, we propose a real-time monitoring system that utilizes IoT-based sensors, big data processing, and a hybrid prediction model for the automotive industry. The proposed IoT-based sensor collects temperature, humidity, accelerometer, and gyroscope data from the assembly line process while the big data processing platform handles and stores the large amounts of generated sensor data. Finally, the proposed hybrid prediction model, which consists of DBSCAN-based outlier detection and Random Forest classification, is used to remove outlier data and provide fault detection during the manufacturing process, respectively.

The remainder of this paper is organized as follows. Section 2 presents a literature review. In Section 3, the methodology is explained, while in Section 4, the results and discussions are presented. In Section 5, concluding remarks are presented and several limitations and remaining challenges are discussed.

## 2. Literature Review

### 2.1. IoT-Based Sensor for Monitoring System

Recent technologies such as IoT, sensors, big data, and machine learning can be utilized for monitoring and can play important roles in predicting disease, improving production, reducing cost, providing an early warning system, and facilitating better decision making for management. Several studies have been conducted on IoT-based monitoring systems and showed significant advantages. Mora et al. proposed an IoT-based framework for monitoring human vital signs [7]. A case study on monitoring footballers’ heart rates during a football match was conducted. The proposed system was able to monitor the players’ vital signs and predict, not only the worst situation (i.e., sudden death), but also possible injuries. Zhang et al. proposed a monitoring system based on IoT for the agricultural field [8]. The developed system was used to monitor citrus soil moisture and nutrients for fertilization and irrigation decision making. Case study-based results showed that the proposed system helped farmers make better decisions, improve citrus production, and reduce labor costs as well as the pollution caused by chemical fertilizers. Manes et al. proposed a distributed monitoring system for leakage detection and gas levels in hazardous environments [9]. A wireless sensor network was utilized to gather the sensor data. The collected environmental sensor data were transmitted to a remote server and presented to the manager via a user interface. The proposed system was effective for monitoring the environment and triggered a warning when critical events were detected. Finally, Cheung et al. proposed real-time monitoring based on information modeling and a wireless sensor network to monitor the safety of construction sites [10]. Hazardous gas levels and environmental conditions (i.e., temperature and humidity) were collected by wireless sensor nodes and sent to a remote server. The proposed system triggered a warning/alarm once an abnormal situation was detected. Case-study based results showed that the proposed system improved the safety of the construction site and helped the management with better decision making in real-time.

Current studies utilize IoT-based sensors to determine the environmental conditions at a real site, thus the sensor data can be presented in real-time. IoT-based sensors provide an important solution for many research areas, including smart building and healthcare. Several studies have been conducted and showed significant results for IoT based sensors on improving system performance. Plageras et al. proposed a monitoring system using IoT-based sensors for smart buildings [55]. The proposed system was implemented in a simulation environment. The results showed that a better monitoring system in a smart building can be achieved by using several IoT-based sensors. The proposed system is expected to improve energy efficiency as well as facilitate green smart buildings. Blanco-Novoa et al. proposed an IoT-based sensor for monitoring the radon gas level inside a building [56]. The proposed system could notify/warn users when a specific level of radon gas is reached to prevent dangerous situations. The proposed system was able to monitor the radon gas level, trigger the programmed actions, and notify the users once a specific level of gas radon was reached. Benammar et al. proposed a modular indoor air quality monitoring system that collects several types of sensor data such as CO_2_, CO, SO_2_, NO_2_, O_3_, Cl_2_, temperature, and humidity [57]. A single-board computer (Raspberry Pi) was utilized as a gateway to process the sensor data. The experimental results showed that the proposed system effectively monitored the indoor air quality for six kinds of gases in addition to temperature and humidity. Sood and Mahajan proposed a healthcare system based on wearable IoT-based sensors for detecting and preventing the outbreak of chikungunya virus [58]. The collected health, environmental, medical, location, and meteorological data were used to classify individuals as possibly infected or uninfected. The results showed that the proposed system could be used to detect infected individuals as well as to send a warning alert to the nearest governments and healthcare clinics to prevent further outbreaks. Finally, Bayo-Monton et al. developed an IoT-based sensor utilizing Arduino and Raspberry Pi to enhance eHealth care [59]. The performance of the proposed sensor was compared with that of a personal computer. The results confirmed that the proposed IoT-based sensor was suitable for scalable eHealth systems.

Several studies have been conducted in the manufacturing industry and showed significant advantages from IoT based sensors in improving working conditions, preventing erroneous designs, providing fault diagnosis and quality prediction, and helping managers with better decision making. Moon et al. developed an IoT-based sensor to measure the air quality inside a factory [11]. Temperature, humidity, CO_2_ level, dust, and odor sensor data were collected and transmitted via wireless communication. Based on the experimental results, the proposed system is robust enough, able to accurately measure the environmental condition in the factory in real-time, and is expected to help managers maintain an optimum working environment for the workers inside the factory. Salamone et al. proposed an environmental monitoring system based on low-cost IoT sensors for preventing errors during the design phase in additive manufacturing [12]. The sensors were used to gather temperature and humidity data. The study revealed that knowledge of environmental conditions could help prevent errors during the design phase in additive manufacturing. Li et al. utilized IoT sensors to collect data for the fault diagnosis of mine hoisting equipment [13]. The study revealed that IoT sensors can help provide complete diagnosis data as well as improve diagnosis results. Lee et al. proposed a framework by utilizing IoT and machine learning to predict the quality of a product and optimize operation control [14]. Metal casting was used as a real-case implementation of the proposed system. The proposed system was able to effectively predict the quality of the metal casting and efficiently improve the operation control. Finally, Calderón Godoy et al. proposed the integration of sensors and the SCADA system for implementation of the fourth industrial revolution framework [15]. Experimental results confirmed the feasibility of the proposed system, which is expected to help managers during the migration of legacy systems to the Industry 4.0 framework.

The number of IoT-based sensors and other related components is increasing significantly. The adoption of IoT in manufacturing enables the transition from traditional to modern digitalized manufacturing. As the number of devices collecting sensor data in manufacturing increases, the potential for new types of applications that can handle the input of large amounts of sensor data such as big data technology also increases. Ge et al. developed a conceptual framework by integrating big data technology in IoT, which is expected to support critical decision making [60]. By utilizing big data processing, the enormous amount of data collected by many heterogeneous sources (sensor devices) can be handled and presented in an efficient manner, thus they can assist managers with better decision making.

### 2.2. Big Data Processing

With the increasing number of IoT and sensing devices, data generated from manufacturing systems are expected to grow exponentially, producing so called “big data” [16]. Big data is often described in terms of 4 V’s. The first V is volume in reference to the size of the data, the second V is variety in reference to the different types/formats of the data, the third V is velocity in reference to the speed of data generation, and the last V is veracity in reference to the reliability of the data [61]. The data generated during manufacturing is increasing daily with different types and formats (i.e., process logs, events, images, and sensor data), hence, the processing and storage of these data is becoming a challenging issue that needs to be addressed. There are several applications of big data analytics in the manufacturing industry. Zhang et al. proposed a big data framework for reducing energy consumption and emission in an energy-intensive manufacturing industry [17]. The proposed system consists of two components, data acquisition for gathering the energy data and data analytics for analyzing the energy usage. Based on a real-case implementation, the results showed that the proposed system was capable of eliminating three percent of the energy consumption and four percent of energy costs. Zhong et al. proposed a big data system for logistics discovery from RFID-enabled production data for mining knowledge [18]. An experiment was used to demonstrate the feasibility of the proposed system and the results showed that the knowledge gained from big data could be used for production scheduling and logistics planning. Mani et al. studied the application of big data analytics for mitigating supply chain social risk [19]. A case study was used to elaborate the application of big data analytics in the supply chain. The results of the study revealed that big data analytics can help management predict various social problems and mitigate social risks. Finally, Li et al. proposed a big data framework for active sensing and processing of complex events in manufacturing processes [20]. To effectively process complex event big data, a relation model and unified XML-based manufacturing processes were developed. The Apriori frequent item mining algorithm was used to find a frequent pattern from the complex events data. The feasibility and effectiveness of the proposed system was confirmed with implementation in a local chili sauce manufacturing company. The proposed model is expected to provide practical guidance for management decision-making.

Several big data technologies can be utilized in the manufacturing industry to process and store large volumes of data quickly, such as Apache Kafka, Apache Storm, and NoSQL MongoDB. Apache Kafka is a scalable messaging queue system used for building real-time applications [62]. It is fault-tolerant, high-throughput, and scalable. Several studies have shown significant benefits from using Kafka for healthcare, transportation, manufacturing, and IoT-generated sensor data. Alfian et al. proposed real-time data processing for monitoring diabetic patients [21]. Apache Kafka and MongoDB were utilized to handle and store sensor data from the patients. The proposed system was sufficiently efficient at monitoring diabetic patients. Ji et al. proposed a cloud-based car parking system consisting of several technologies, including Apache Kafka [63]. The proposed system was capable of efficiently handling massive amounts of sensor data when the amount of data and the number of clients increased. D’silva et al. proposed a framework for handling real-time IoT event data [22]. The proposed framework utilized Apache Kafka as a message queue system and was efficient enough to process real-time IoT events data. Canizo et al. proposed a framework based on big data technologies and machine learning for online fault prediction for wind turbines [23]. Apache Kafka was used to handle incoming data in real-time and send the data to a streaming system for further analysis. The proposed system could be used to monitor the status of wind turbines and is expected to help reduce operation and management costs. Du et al. proposed a framework for handling huge amounts of incoming unstructured connected vehicle (CV) data [24]. The proposed framework utilized Apache Kafka as a distributed message broker. Experimental results showed the proposed system is efficient enough in handling huge amounts of incoming CV data and achieved the minimal recommended latency value defined by the U.S. Department of Transportation for CV applications. Park and Chi proposed an architecture for an ingestion system based on Apache Kafka for machine logs in the manufacturing industry [25]. The proposed system collects machine logs from a set of milling machines, handles them in a Kafka messaging queue, and delivers them to an external systems for further analysis. Finally, Ferry et al. proposed a data management system based on big data technologies for machine generated data in a manufacturing shop-floor [26]. The proposed system utilizes Apache Kafka as a message queue and Apache Storm as a real-time processing system. Implementation of the proposed system is expected to reduce infrastructure and deployment costs.

Apache Storm is a real-time distributed parallel system for processing high-velocity stream data [64]. It is fault-tolerant and scalable, with guaranteed data processing. Previous studies have utilized Apache Storm for real-time data processing. Ma et al. proposed a stream-based framework for providing real-time information services on public transit [27]. The proposed framework utilized Apache Storm as a real-time distributed processing engine. The results showed that the proposed framework was capable of handling large amounts of real-time data with lower latency. Furthermore, the performance of the proposed framework increased when the number of nodes/servers utilized increased. Manzoor and Morgan proposed a real-time intrusion detection system based on Apache Storm [28]. The proposed system was evaluated using the KDD 99 network intrusion dataset and the results showed that the proposed system was feasible for processing network traffic data and detecting network intrusion with high accuracy. Chen et al. proposed a real-time geographic information system for managing environmental big data using Apache Storm [29]. The proposed system was tested with two use-cases (i.e., real-time air quality monitoring and soil moisture monitoring). The results showed that the proposed system was effective enough for managing real-time environmental big data. In addition, several studies have been conducted regarding the performance of Apache Storm as a real-time data processing system. Qian et al. performed a performance comparison between Apache Storm and Spark [30]. The latency and throughput of the system was considered and the results showed that Apache Storm has shorter latency while Spark has higher throughput. Finally, Chatterjee and Morin performed comparative performance analysis between several data streaming platforms (i.e., Flink, Storm, and Heron) [31]. Various performance metrics were considered such as fault tolerance and resource usage. The results showed that Storm has better fault tolerance and less memory usage than the other systems.

The increasing amount of IoT-generated sensor data has led to increased demand for sensor-friendly data storage platforms. NoSQL databases have become popular in the last couple of years because of their growing flexibility, scalability and availability. The term ‘NoSQL’ collectively refers to data storage platforms that do not follow a strict data model for relational databases. MongoDB is a document-oriented NoSQL database that offers flexible data-schema, high performance, scalability, and availability [65]. A previous study compared the performance of MongoDB and Oracle with insert, update, and delete tests [32]. MongoDB outperforms oracle in all tests. In addition, MongoDB has been proven to be effective for storing data from the supply chain, geographic information systems and manufacturing. Alfian et al. utilized MongoDB to store IoT-generated sensor data for monitoring a perishable food supply chain [33]. In the study, MongoDB was capable of processing a huge amount of input/output sensor data efficiently when the number of sensors and clients increased. In addition, MongoDB outperformed MySQL in read and write tests. Hu et al. conducted a comparative study among six popular databases (i.e., Rasdaman, SciDB, Spark, ClimateSpark, Hive, and MongoDB) for handling a variety of geospatial data [34]. The results showed that MongoDB was adequate in terms of parallel query and resource consumption (i.e., CPU, memory, network). Chen et al. proposed MongoSOS, a sensor observation service based on MongoDB, for handling spatiotemporal data [35]. The proposed system was capable of handling read and write access for navigation and positioning data in a millisecond and the performance improved by around two percent compared with the traditional model. Putri et al. proposed a big data processing system based on Apache Spark and MongoDB to identify profitable areas from large amounts of taxi trip data [36]. The experimental results showed that the proposed system was scalable and efficient enough in processing profitable-area queries from huge amounts of big taxi trip data. Finally, Angrish et al. proposed a flexible data schema based on NoSQL MongoDB for the virtualization of manufacturing machines [37]. The proposed system was evaluated against several query statements. The results showed that MongoDB can accommodate any type of machine data and could easily be implemented across a variety of machines on the factory floor.

Previous studies have shown a significant impact from the integration of several big data technologies. Lohokare et al. proposed a scalable framework for home automation in smart cities [38]. The proposed framework utilized Apache Kafka as a message broker to handle incoming IoT data and MongoDB to store the sensor data. The proposed system was able to reduce the processing time when the amount of data and nodes increased. Jung et al. proposed a smart city system using Apache Kafka and Apache Storm to handle and process IoT-generated data in real-time [39]. Experimental results showed that the proposed system was capable of effectively and efficiently processing the IoT-generated data in real-time. Villari et al. proposed a management system for smart environments using big data technologies [40]. The proposed system utilized Apache Storm to process the data in real-time and MongoDB to store huge amounts of sensor data. A case study on smart homes was performed, and the results showed that the proposed system was able to manage large amounts of smart environmental data in real-time. Zhou et al. proposed an integration of Apache Kafka, Apache Storm, and MongoDB for processing streaming spatiotemporal data [41]. The proposed system was tested using the Taiyuan BeiDou bus location data. The proposed system was capable of processing large amounts of sensor data per second and was around three times faster than the traditional model. Finally, Syafrudin et al. proposed an open source-based real-time data processing system consisting of Apache Kafka, Apache Storm, and MongoDB [42]. The proposed system was implemented to monitor the injection molding process in real-time. The proposed system was capable of processing a massive amount of sensor data efficiently when the amount of data and the number of devices increased.

Integration of Apache Kafka, Apache Storm, and MongoDB can be used for big data processing to handle manufacturing sensor data. Previous studies have shown that these three technologies can be used for big data processing so that large amounts of streaming sensor data can be promptly processed, stored, and presented in real-time [41,42]. Thus, in our study, Apache Kafka, Apache Storm, and MongoDB were utilized for big data processing to monitor the manufacturing process in real-time. In addition, the integration of big data processing with a machine-learning model is expected to help managers with decision-making and to prevent unexpected losses caused by faults during the manufacturing process.

### 2.3. Machine Learning Methods in Manufacturing

The manufacturing industry is experiencing an increase in data generation, e.g., sensor data from the production line, environmental data, etc. New developments in technology such as machine learning offer great potential to analyze data repositories, and thus can provide support for management in decision-making or can be used to improve system performance. Machine learning techniques are utilized to detect certain patterns or regularities and have been successfully implemented in various areas such as fault detection, quality prediction, defect classification, and visual inspection. Several studies have utilized machine learning and showed significant results in the manufacturing industry. Kim et al. employed seven different machine learning-based novelty detection methods to detect faulty wafers [43]. The models were trained with Fault Detection and Classification (FDC) data to detect faulty wafers. The experimental results showed that machine learning-based models had a high possibility of detecting faulty wafers. Lee et al. performed an evaluation analysis on four machine learning algorithms (i.e., decision tree, random forest, artificial neural network, and support vector machine) for predicting the quality of metal castings product [14]. The result showed that all of four machine learning algorithms can effectively be used to predict the quality of product. Chen et al. utilized support vector machine algorithm to predict the quality of welding in a high-power disk layer [44]. The results showed that the proposed quality prediction model can be used for real-time monitoring system. An intelligent system was developed by Chen et al. to minimize the incorrect warning in detecting the quality of product in manufacturing [45]. They utilized three methods (i.e., visual inspection, support vector machine, and similarity matching). Through real-case implementation in manufacturing company in Taiwan, the proposed system can effectively be used to minimize the incorrectly classified and improve the performance of quality prediction. Finally, two machine learning algorithms (i.e., decision tree and Naïve Bayes) was also used by Ravikumar et al. for automating the process of inspecting the quality of machine components [46]. Three types of machine component quality (i.e., good, minor scratch, and deep scratch) were measured. The results showed that the proposed method can effectively be used in automating the quality inspection of the product in real practical case.

Fault detection and diagnosis is an important problem in process engineering and is utilized to detect abnormal events in a process. Early detection of process faults can help avoid productivity loss. Machine learning algorithms such as Random Forest showed significant efficacy in detecting process faults in manufacturing. Random Forest is an ensemble prediction method that aggregates the results of individual decision trees [66]. Generally, Random Forest works by utilizing the bagging method to generate subsets of training data. For each training dataset, a decision tree algorithm is utilized. In the end, the final prediction result is selected based on majority vote (the most voted class) over all the trees in the forest. Recently, Random Forest was used by Quiroz et al. for detecting the failure of rotor bar. They performed the performance analysis between Random Forest and other models (i.e., decision tree, Naïve Bayes, logistic regression, linear ridge, and support vector machine). The experimental results showed that Random Forest outperformed the other models and has around 98.8% of accuracy. The proposed model can be used for real-time fault monitoring system as well as the preventive maintenance system in factory. Random Forest also was utilized by Patel and Giri for detecting the failure of bearing [48]. The results were compared with those obtained from an existing artificial intelligence technique, neural network. The results showed that Random Forest had better performance and higher accuracy than the neural network algorithm. The results of this study are expected be used for bearing fault detection and diagnosis. Finally, Cerrada et al. proposed fault diagnosis in spur gears based on genetic algorithm and Random Forest [49]. The proposed system consisted of two parts, namely genetic algorithm for attribute selection and Random Forest for classification. The proposed system was tested on real vibration signals and Random Forest had better performance for fault diagnosis.

Machine learning algorithms encounter problems with outlier data, which can reduce the accuracy of the classification model. Outlier detection can be utilized in the preprocessing step to identify inconsistencies in data/outliers; thus, a good classifier can be generated for better decision making. Previous studies showed that removing the outlier can improve the classification accuracy. Tallón-Ballesteros and Riquelme utilized outlier detection for a classification model [50]. The authors proposed a statistical outlier detection method based on the interquartile range (lQR) with classes. The results showed that by removing the outliers from the training set, the classification performance of C4.5 was improved. Podgorelec et al. utilized an outlier prediction method to improve classification model performance in medical datasets [51]. The results showed that by removing the identified outliers from the training set, the classification accuracy was improved, especially for the Naïve Bayes classifier.

One of the techniques used for outlier detection is DBSCAN [52]. The algorithm works by identifying dense regions, which are determined based on the number of objects close to a given point. Finally, the algorithm identifies points that do not belong to any cluster, which are treated as outliers. DBSCAN has been implemented in different areas and showed significant accuracy by detecting true outliers. Tian et al. proposed an outlier detection method involving soft sensor modeling of time series [53]. They utilized DBSCAN for outlier detection and the proposed outlier detection method demonstrated good performance. Abid et al. proposed outlier detection based on DBSCAN for sensor data in wireless sensor networks [54]. The proposed model successfully separated outliers from normal sensor data. Based on experiments with synthetic datasets, the proposed model showed significant accuracy in detecting outliers, with an accuracy rate of 99%.

Existing studies showed that Random Forest can be utilized for fault prediction with high classification accuracy. Furthermore, several studies showed significant results for DBSCAN-based outlier detection with regard to improving the classification accuracy. We propose a hybrid prediction model that consists of DBSCAN-based outlier detection to remove the outlier data, and Random Forest to detect whether the manufacturing process is functioning normally or abnormally. The hybrid prediction model is integrated with a real-time big data processing system, enabling processing of the sensor data from IoT-based sensor device (e.g., temperature, humidity, accelerometer, and gyroscope) and fault prediction in real-time.

## 3. Methodology

### 3.1. System Design

**Figure 1:**
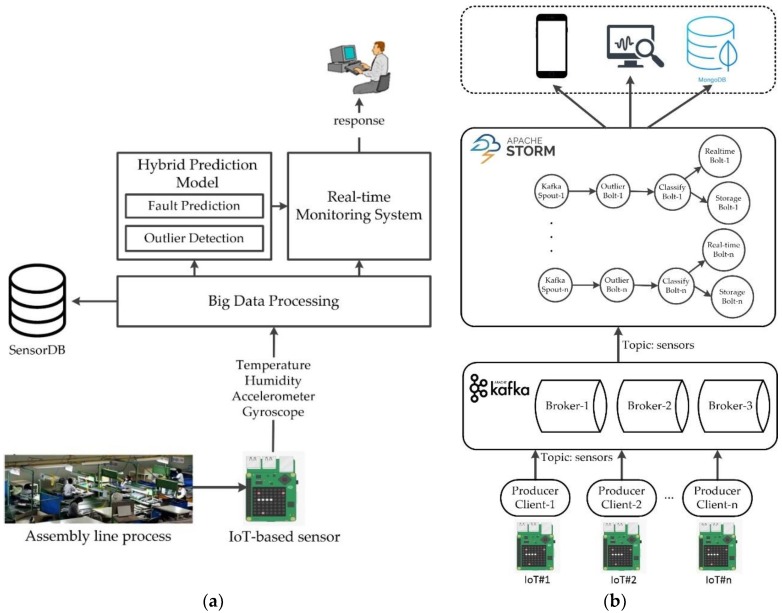
Architecture of the real-time monitoring system in an assembly line process (**a**) and system design for big data processing (**b**).

The real-time monitoring system proposed here was developed to help managers to better monitor the assembly line process in an automotive manufacturing as well as provide early warning when a fault is detected. The proposed system utilizes IoT-based sensors, big data processing, and a hybrid prediction model. The hybrid prediction model consists of clustering-based outlier detection and a machine learning-based classification model. As can be seen in Figure 1a, IoT-based sensors are attached to the desk of a workstation in the assembly line. The IoT-based sensors consist of temperature, humidity, accelerometer, and gyroscope sensors. The IoT-generated sensor data is transmitted wirelessly to a cloud server where the big data processing system is installed. The system allows the system to process large amounts of sensor data quickly before they are stored in the MongoDB database. A clustering-based outlier detection method is utilized to filter out outliers from the sensor data. In addition, a data analytics machine learning-based classification model is applied to predict faults given by the current sensor data during the assembly line process. Finally, the complete history of the sensor data such as the temperature, humidity, accelerometer, and gyroscope data are presented to the manager in real-time via a web-based monitoring system in addition to the fault prediction results.

The proposed big data processing system utilizes Apache Kafka, Apache Storm, and MongoDB. Apache Kafka is a message queue system with low-latency, high-throughput, and fault tolerance, capable of publishing streams of data. Apache Storm is a real-time parallel data processing system with horizontal scalability, fault tolerance, and guaranteed data processing and can process large volumes of high-velocity streams of data. Figure 1b shows the system design for the big data processing system proposed for real-time monitoring. The sensor data from the IoT-based sensor device is wirelessly transmitted using a python-based program developed to serve as the “producer” for the Kafka server. The “producer” client publishes streams of data to Kafka “topics” distributed across one or more cluster nodes/servers called “brokers”. The published streams of data from Kafka are then processed by Storm in parallel and real-time. Outlier detection and classification are implemented inside Storm. The sensor data and the classification results are stored in MongoDB and presented in a web-based monitoring system in real-time.

**Figure 2:**
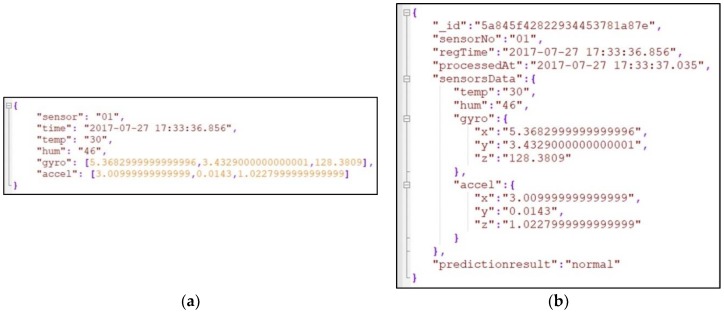
An example of sensor data generated by the IoT-based sensor presented in JSON format (**a**); and when stored in NoSQL MongoDB (**b**).

The characteristics of IoT-generated sensor data are as follows: large amount, unstructured format, and continuous generation. Figure 2a shows an example of the data generated by IoT-based sensors in JSON format before being sent to the Kafka server. The sensor data is delivered to Storm where the hybrid prediction model (i.e., outlier detection and fault classification) is implemented. The sensor data and the prediction results are then stored in NoSQL MongoDB. An embedding scheme-based sensor data repository is commonly utilized in NoSQL MongoDB databases to improve performance [67]. We found that the embedding scheme is appropriate for a large sensor data repository, which requires fast read and write performance [33]. Thus, in our study, we utilized an embedding scheme-based sensor data repository. As can be seen in Figure 2b, the sensor document consists of the ID of the IoT device, the recorded time, processed time, sensor data, and prediction results. The sensor data such as temperature, humidity, gyroscope, and accelerometer data are embedded as a subdocument.

### 3.2. System Implementation

**Figure 3:**
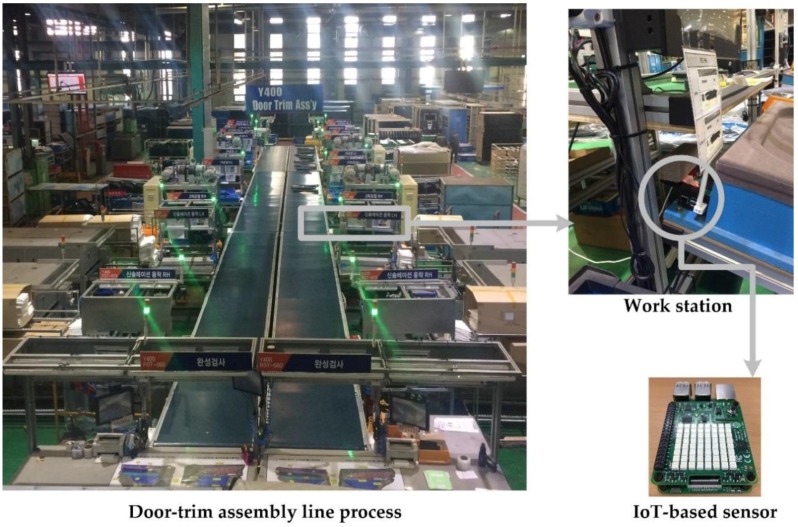
The real-case implementation of the proposed IoT-based sensor in an assembly line.

In this study, the monitoring system was applied to monitor the assembly line process for producing door-trim at an automotive manufacturing in Korea, as shown in Figure 3. The developed IoT-based sensor consists of a Raspberry Pi [68] as the single main board and Sense-HAT [69] as an add-on sensor board. Raspberry Pi is a small single-board computer with the dimensions of 85.60 mm × 53.98 mm × 17 mm, weighing only 45 g, and is affordable at approximately $25–35 USD. It has USB, LAN, HDMI, audio, and video ports for various input and output operations. In addition, general-purpose input-output (GPIO) connectors enable additional devices, or add-on boards such as sensors, to be connected to the main board [70]. The detailed specifications of the Raspberry Pi board can be seen in Table 1. The Sense-HAT board is an add-on sensor board that measures temperature, humidity, accelerometer, and gyroscope data and is designed as an official add-on board for the Raspberry P*i*. The detailed specifications of the Sense-HAT board can be seen in Table 2. The Sense-HAT board is attached to a Raspberry Pi via GPIO 40 pins. The assembled and real-case implementation versions of the IoT-based sensor device can be seen in Figure 3.

In this study, we developed a python-based program as a client using the supplied official application programming interface (API) to gather sensor data from IoT-based sensors [71]. The IoT-based sensors continuously collect temperature, humidity, gyroscope, and accelerometer data, which are transmitted to a cloud server wirelessly. As can be seen in Figure 3, an IoT-based sensor device is attached to the desk of a workstation panel along the assembly line process. The IoT-based sensor senses the environmental conditions and sends the sensor data to a cloud server every 5 s. The sensor data are processed by the big data processing system and analyzed further in real-time. Finally, the historical sensor data are saved in MongoDB and presented on a web-based monitoring system in real-time.

### 3.3. Hybrid Prediction Model for Fault Detection

**Figure 4:**
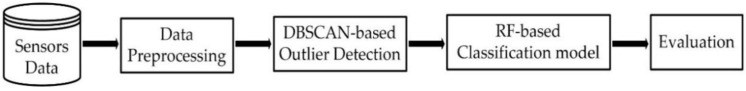
Hybrid Prediction Model using Density-Based Spatial Clustering of Applications with Noise (DBSCAN)-based outlier detection and Random Forest (RF)-based classifier.

In this study, the hybrid prediction model is utilized to predict whether the process is functioning normally or abnormally. Figure 4 shows the process of detecting normal or abnormal events during the manufacturing process. The hybrid prediction model utilizes an outlier detection based on DBSCAN to detect and remove outliers from the sensor data and a Random Forest-based classification model to predict normal and abnormal events. Finally, the performance is evaluated by comparing the hybrid prediction model with other classification models.

For the performance evaluation of various prediction models, the dataset was collected from experiments in a lab in which the IoT-based sensor was installed. The collected dataset consisted of 342 instances, which were classified as normal or abnormal events during the manufacturing process. The dataset contained eight features: (1) temperature (°C), (2) humidity (% relative humidity/rh), (3) the X value of the accelerometer, (4) the Y value of the accelerometer, (5) the Z value of the accelerometer, (6) the X value of the gyroscope, (7) the Y value of the gyroscope, and (8) the Z value of the gyroscope. The dataset consisted of 102 data points labeled as “yes” and 240 labeled as “no”. A “yes” class indicates an abnormal event occurred while a “no” class means an abnormal event did not occur during the manufacturing process (normal). In addition, the collected training dataset (342 instances) was labeled based on the possible combination of fault events during assembly line process in automotive manufacturing. The machine learning methods are expected to learn and generate the robust model/classifier from collected dataset. Once the model/classifier is generated and installed into monitoring system, the prediction result from real-time IoT-based sensor data can be presented.

Once the dataset was collected, data preprocessing was performed by removing inappropriate, inconsistent, and missing-value data. Table 3 shows the dataset distribution for the mean and standard deviation of each class. Furthermore, in order to analyze the significance of the features, the Information Gain (IG) technique was applied [72]. Weka version 3.6.15 software was utilized to evaluate the significance of the features with IG [73]. The dataset attributes and their IG scores are presented in Table 4. The results show that temperature is the greatest factor that affects abnormal events during the manufacturing process.

DBSCAN-based outlier detection was utilized in our study to filter out outlier data from the dataset [52]. Dense regions were created by finding the objects close to a given point. Outliers were defined as the points located outside dense regions. Epsilon (*eps*) and minimum points (*MinPts*) are two important parameters considered in DBSCAN. *eps* defines the radius distance of the neighborhood around a point x (*ϵ-neighborhood* of x) and *MinPts* defines the minimum number of neighbor points within the defined radius distance of *eps*. For dataset *D*, which is marked as unvisited, DBSCAN works as follows:

- For each unvisited point $x_{i}$ in *D*, find the *ϵ-neighborhood* of $x_{i}$ that includes at least *MinPts* points. Then $x_{i}$ is labeled as visited.
- For point $x_{i}$, which is not assigned to a specific cluster, create a new cluster *C*. Add the points in the *ϵ-neighborhood* of $x_{i}$ to a candidate set *N*. Add any points in *N* (that do not belong to any cluster) to *C*.
- For each point *p* in *N*, find the *ϵ-neighborhood* of *p* that includes at least *MinPts* points. Those points in the *ϵ-neighborhood* of *p* are then included in the candidate set *N* and assigned to cluster *C*. Finally, *p* is labeled as visited.
- Iterate the process for the remaining points in *N* and the unvisited points in the dataset *D*.
- The points that do not belong to any cluster are labeled as outliers.

**Figure 5:**
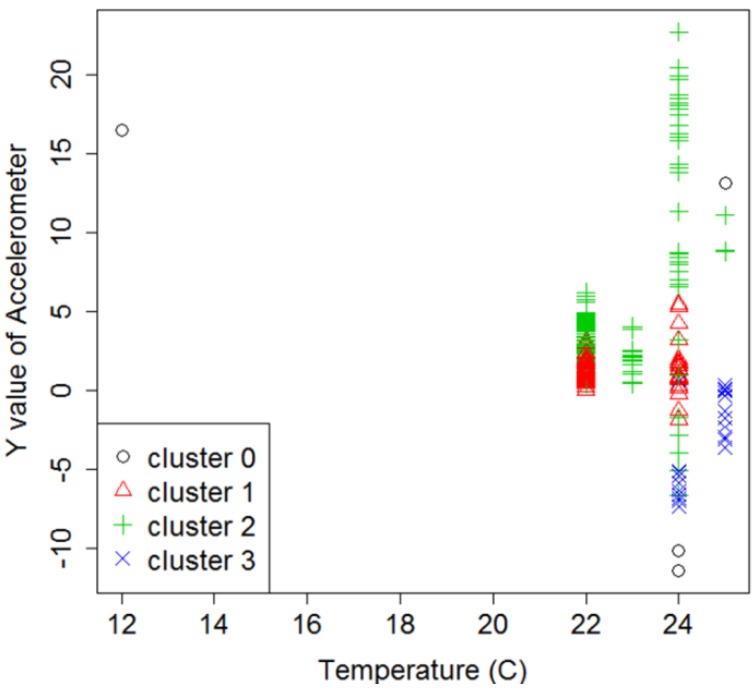
The result of DBSCAN-based outlier detection.

Due to the imperfect sensing device and network connection problems, some of the data collected by the sensor may be noise caused by outlier data. Outlier detection based on DBSCAN was applied to our dataset. The optimal value of *MinPts* and *eps* should be defined first in order to perform DBSCAN-based outlier detection. If the value of *eps* is too small, more clusters will be created, and normal data could be classified as outliers. However, if it is too big, less clusters will be generated, and true outliers could be classified as normal data. Through the different setup of the experiments, the optimal parameters for *MinPts* and *eps* were discovered, they are 5 and 7. Figure 5 shows the results of DBSCAN implementation for the dataset in two-dimensional graphs. DBSCAN performed clustering by grouping the data into three clusters, presented as clusters 1, 2, and 3. The outliers were unclustered data and were presented as cluster 0. The description of dataset, optimal parameters, and outlier data are presented in Table 5. Finally, the outlier data were removed from the dataset, and the remaining data were used for further analysis.

Random Forest is a popular classification method for solving real-world classification problems [66,74,75,76]. The Random Forest algorithm is constructed by combining multiple decision trees for more accurate and stable prediction [77]. Every tree inside a Random Forest is independently constructed by selecting a random subset of features and bootstrap sampling of the dataset. Next, the tree is grown to the largest possible level. Each decision tree model inside the Random Forest will generate a prediction output and a majority vote is applied to obtain the final prediction output. Majority vote is a well-known method to obtain a better final prediction output [77]. Previous studies have utilized Random Forest because of its robustness when dealing with numerical data and solving real-world problems [74,75,76]. Recently, Random Forest was utilized for predicting the crash of stopping maneuvering [76]. The results showed that Random Forest successfully detected the crash of stopping maneuvering and forecast the safety properties of the ship before production. In our study, DBSCAN-based outlier detection was utilized to remove outlier data from the dataset and Random Forest was utilized to learn from the training set. Finally, the results of prediction were compared with the testing set to determine the model accuracy.

Based on a confusion matrix [78], the prediction output can have four possible outcomes, as can be seen in Table 6. True positive (TP) and true negative (TN) results are defined as the number of correctly classified points. False positive (FP) and false negative (FN) results are defined as the number of points incorrectly classified as “yes” (positive) when they are actually “no” (negative) and incorrectly classified as no (negative) when they are actually yes (positive), respectively. In our dataset, abnormal events during the manufacturing process were defined as “Yes” and normal events were defined as “No”. For training and testing the dataset, 10-fold cross-validation was applied for all classification models. The final performance measure was obtained by averaging the test performance for all folds. Weka Software 3.6.15 was utilized to run the classification models for the dataset [73]. Table 7 shows the measured performance metrics for the classification model based on precision, recall/sensitivity, and accuracy.

## 4. Results and Discussions

### 4.1. Real-Time Monitoring System

**Figure 6:**
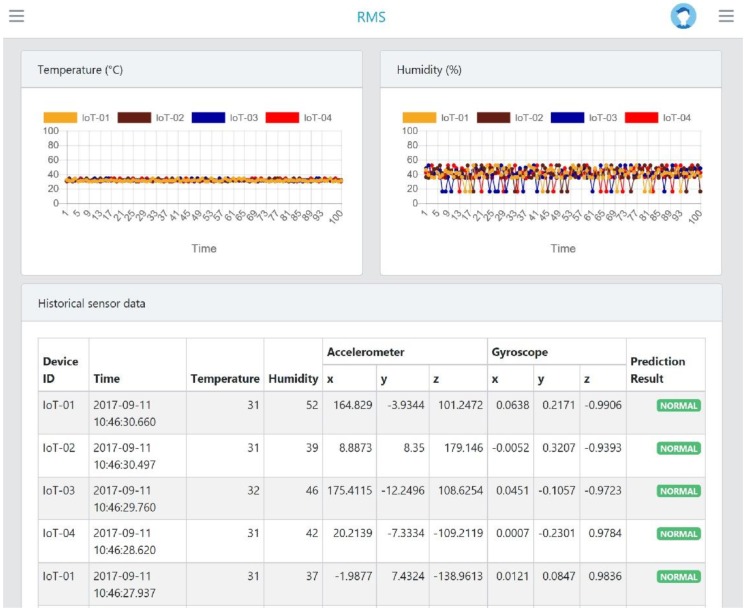
The web-based real-time monitoring system.

Data visualization was developed by utilizing JavaScript framework as a monitoring system to present sensor data in real-time. The manager could monitor the status of assembly line process as well as receive the early warning once the abnormal event (fault) is detected in real-time through the proposed system. The IoT-based sensor devices sent the sensor data to Apache Kafka, then Apache Storm will process the data as well as sent the sensor data and its fault prediction results directly to the monitoring system in real-time, and finally the sensor data and its prediction result are stored into MongoDB. As can be seen in Figure 6, the real-time monitoring system can be easily accessed via a web-browser on a personal computer. The proposed system presents the sensor data such as temperature, humidity, accelerometer, and gyroscope data in real-time. The device ID (IoT-based sensor device) and recorded time was collected and presented for every record. In addition, the hybrid prediction model was used to predict the fault and present the result into real-time monitoring system. The proposed system has been implemented and tested in one of automotive manufacturing in Korea from 1 August 2017 to 31 March 2018. Four IoT-based sensor devices were installed in the manufacturing assembly line and transmitted the sensor data to the remote server every 5 s. During this testing period, around 19 million records (with approximate size is 3 gigabytes) has been collected. Our proposed real-time monitoring system consists of three parts: the IoT-based sensor, the big data processing platform and hybrid prediction model. The performance evaluation are presented for each part in Section 4.2, Section 4.3 and Section 4.4, respectively.

### 4.2. Performance of the IoT-Based Sensor

An IoT-based sensor consists of a sensor device and a client program to retrieve sensor data and send them to a cloud server. It is important to analyze the IoT-based sensor performance under various conditions. Performance metrics such as network delay and CPU and memory usage were utilized in this study. Alazzawi and Elkateeb proposed network delay as a metric to evaluate the sensor device performance [79], while Morón et al. utilized CPU usage as a metric to evaluate IoT device capabilities in different scenarios [80]. In our study, network delay was defined as the average time between sending sensor data from by the source (sensor device) and successfully receiving the data at the destination (MongoDB). The second performance metric was the average CPU and memory usage of the client program under various scenarios.

**Figure 7:**
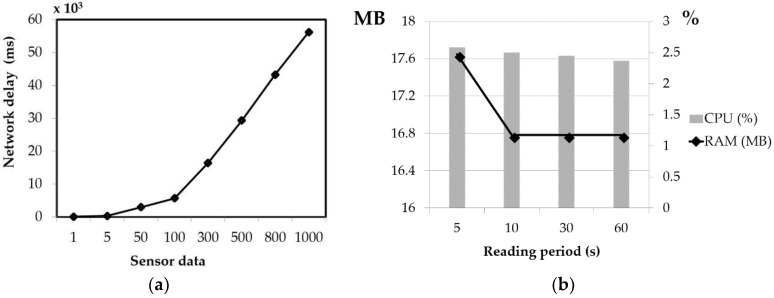
The IoT-based sensor system’s (**a**) network delay, and (**b**) CPU and memory usage.

In this study, the client program was a python-based program running on an IoT-based sensor device that collected sensor data such as temperature, humidity, gyroscope, and accelerometer data. An IoT-based sensor with Linux Raspbian OS Jessie; 1 GB RAM was used for the experiment. Communication between the IoT-based sensor and cloud server was implemented via Wi-Fi. Figure 7a shows the network delay for different amounts of sensor data. The results show that the network delay increases as the amount of sensor data sent by the sensor device increases. It takes approximately 50 s for the IoT-based sensor to send 1000 sensor data points at the same time. However, in a real-case implementation, it takes less than 0.02 s to send the sensor data, as we only set one sensor data point (temperature, humidity, gyroscope, and accelerometer data) to be sent every 5 s. In addition, Figure 7b shows the CPU and memory usage of the client program. Four different reading period scenarios were evaluated, in which the client program was reading and sending sensor data to the cloud server every 5, 10, 30, and 60 s. The results showed that the reading period has a very small effect on CPU or memory usage. Regarding the computational cost of the client program, it should be noted that the program used less than 3% CPU and 18 MB for all reading periods.

### 4.3. The Performance of Big Data Processing

It is important to analyze the performance of big data processing under various conditions. Performance metrics such as system latency, throughput, and concurrency were utilized in this study. Pereira et al. utilized system latency and throughput to evaluate the performance of big data technology under different operations [81], while Van der Veen et al. used concurrency to evaluate big data technology under multiple clients [82]. In our study, system latency is defined as the time needed by the proposed system to handle, process, and store the sensor data into database. Throughput is defined as total number of sensors data processed per second. The last metric is concurrency which is defined as the number of clients accessed simultaneously to the system. The experiments was conducted with different numbers of servers and the response time was collected for analysis. The Java program was developed as a simulator to generate sensor data and send the data to the big data processing servers. The server was installed with Apache Kafka, Apache Storm, and MongoDB. The threads was used by Java program to simulate multiple clients. The detailed specifications of client and server computer can be seen in Table 8. In addition, the approximate size of each simulated data is around 211 bytes which consists of the device ID, the date and time when the data is generated and the value of sensor data (temperature, humidity, accelerometer, and gyroscope).

**Figure 8:**
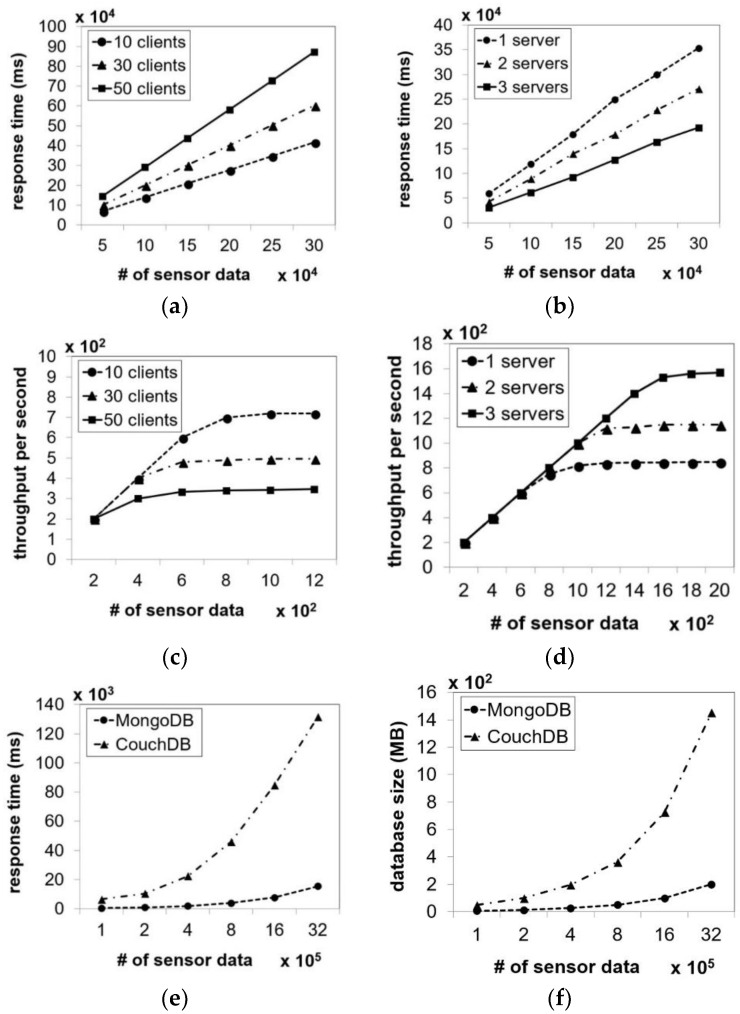
Performance evaluation in terms of latency with different numbers of clients (**a**) and servers (**b**); throughput with different numbers of clients (**c**) and servers (**d**); comparison between MongoDB and CouchDB databases in terms of latency (**e**); and database size (**f**).

Figure 8a shows that as the amount of sensor data sent to the cloud server increased, the response time also increased. The number of clients also affected the response time, since more time was required for the proposed system to process and store sensor data sent by a larger number of clients simultaneously. However, taking advantage of scalability support by adding more servers can help achieve lower response time compared to a single server as shown in Figure 8b. Figure 8c,d show the system throughput with different numbers of clients. Better performance could be achieved by increasing the number of servers. Furthermore, Figure 8e,f compare the system latency and database size of MongoDB and CouchDB. In this test, we used a single client and sent different amounts of sensor data to the cloud server at the same time. The Java Program was implemented on the client-side to send the sensor data to the cloud server. MongoDB performed better than CouchDB when the amount of sensor data increased. In addition, MongoDB occupied a lower database size than CouchDB did.

### 4.4. Hybrid Prediction Model for Fault Detection

During dataset generation, the big data processing system receives the sensor data from the IoT-based sensor device and stores the data in NoSQL MongoDB. The IoT-based sensor collects data from different types of operation, including normal and abnormal events. The dataset is then labeled by expert users based on the process status (either normal or abnormal) during the period when the sensor data were collected. Next, the dataset is analyzed using the hybrid prediction model to predict the fault status. The performance comparison results for several classification models are presented in Table 9. Several conventional classification models such as Naïve Bayes (NB), Logistic Regression (LR), Multilayer Perceptron (MLP), and Random Forest (RF) were compared with the hybrid prediction model to identify and predict abnormal events. The proposed model achieved the highest accuracy (100%) compared to other classification models. There was slight improvement in model accuracy after the implementation of DBSCAN-based outlier detection. Integrating DBSCAN-based outlier detection with the Random Forest model increased the accuracy by as much as 1.462% compared to conventional Random Forest. Furthermore, the accuracy improvement has been found in other conventional classification models after applying DBSCAN for outlier detection as much as 3.173%, 0.567%, and 2.026% for Naïve Bayes, Logistic Regression, and Multilayer Perceptron, respectively.

The proposed model was implemented in Apache Storm where the streams of data from Kafka can be processed and predicted in parallel and real-time. Figure 6 shows the results of implementation where real-time prediction is performed by Apache Storm to identify whether the process is functioning normally or abnormally given the input data from the IoT-based sensor (e.g., temperature, humidity, accelerometer, and gyroscope). The results of the study are expected to help management prevent unexpected losses caused by faults at an early stage and improve decision-making during the manufacturing process.

### 4.5. Managerial Implications

In this study, the proposed system consists of three parts: the IoT-based sensor, big data processing, and machine learning model. First, the IoT-based sensor device developed in this study is based on Raspberry Pi which is small-size, low-cost, and powerful single-board computer device. Previous studies have shown significant advantages of utilizing Raspberry Pi such as for controlling and monitoring IoT system [83], estimating the roll angle of a vehicle using embedded neural network in real-time [84], hosting and serving the user interface of eHealth care system [59], and monitoring the temperature of lava lake using near infrared thermal camera [85]. Therefore, the proposed IoT-based sensor device developed in this study could be applied to monitor the manufacturing process in real-time. Second, since the number of IoT devices increased, it is necessary to develop new big data processing to effectively handle, process, and store the data without experiencing detectable performance loss. Previous studies revealed that by implementing open source software (OSS), the organizations can achieve some economic gains in terms of software development productivity, product quality, as well as lower cost (i.e., license costs) and availability of external support [86,87]. In our study, the developed big data processing platform is based on the OSS that is cost-effective for implementation and integration. Third, machine learning has been used in various processes for monitoring systems in manufacturing and predictive maintenance in different industries [88,89,90,91,92]. Machine learning has powerful tools for continuous quality improvement in a large and complex process such as semiconductor manufacturing [89,90,92]. In our study, the machine learning model is used to detect the fault (abnormal event) during assembly line process in real-time. Thus, it is expected to support the management in improving the decision-making and preventing the unexpected loss caused by faults at an early stage during manufacturing process. Finally, the overall results of the study can be used as a guideline for the industrial practitioner in adopting the IoT, big data, and machine learning for their manufacturing process.

Previous scholars and practitioners have considered several aspects of big data. Big data is often described in terms of 4 V’s, they are volume (the size of data), variety (different type of data), velocity (speed of data generation), and veracity (reliability of data) [61]. However, some scholars are more focused on one or more aspects of the big data concept. Davenport et al. focused more on the variety aspect of data sources [93], while some other authors emphasized the storage (volume) and analysis parts when it comes to dealing with big data [94,95]. The big data processing that efficiently can handle the fast incoming (velocity) and huge amount (volume) of sensor data has been developed in our study. Finally, the integration of an IoT-based sensor, big data processing, and machine learning model can be utilized to effectively monitor the manufacturing process as well as obtain early warning notification when an abnormal event is detected in real-time.

## 5. Conclusions

In this study, we developed a real-time monitoring system that utilizes IoT-based sensors, big data processing, and a hybrid prediction model. The proposed model is expected to help managers monitor the status of the assembly line process and to identify faults in the process, thus unexpected losses caused by faults can be prevented. Through this study, we showed that integrating IoT-based sensors with a big data processing system is effective for processing and analyzing large amounts of sensor data in real-time. The big data processing system developed in this study utilizes Apache Kafka, Apache Storm, and NoSQL MongoDB. The experimental results showed that the system is scalable and can process a large amount of continuous sensor data more efficiently than traditional models. Furthermore, the performance of the IoT-based sensor was analyzed with various metrics such as the network delay, CPU, and memory usage. For all experimental scenarios, the IoT-based sensor provided an efficient solution as it successfully collected and transmitted the data within an acceptable time with low computational cost.

Fault detection is an important issue in the manufacturing process as it can identify whether the process is functioning normally or abnormally. We propose a hybrid prediction model that consists of DBSCAN-based outlier detection and Random Forest classification. DBSCAN was used to separate outliers from normal sensor data, while Random Forest was utilized to predict faults—given the sensor data as input. The results showed that the proposed hybrid prediction model is effective with high accuracy compared to the other models tested. The results of the study are expected to support management and improve decision-making during manufacturing, helping prevent unexpected losses caused by faults.

Security is a big issue when more IoT devices are adopted, implemented, and connected. Therefore, the security of IoT devices and platforms should be considered in a future study. Furthermore, a variety of abnormal conditions during the manufacturing process should be further identified and collected so the proposed hybrid prediction model can be utilized to learn from a complex dataset in the near future.

## Figures and Tables

**Table 1:** Detailed specifications of Raspberry Pi 3 model B.

Specification	Information
RAM	1 GB
CPU	Quad Cortex A53 @ 1.2 GHz
GPU	400 MHz VideoCore IV
GPIO	40 pins
Storage	Micro-SD
Ethernet	10/100 Mbps
Wireless	Wireless LAN 802.11n/Bluetooth 4.0 Low Energy
USB	4 ports
Power consumption	5 V
Dimensions	85.60 × 56.5 mm

**Table 2:** Detailed specifications of Sense-HAT.

Specification	Information
Gyroscope	Gyroscope sensor (accurate to ±245/500/2000 degrees per second)
Accelerometer	Accelerometer sensor (accurate to ±2/4/8/16 G-forces)
Magnetometer	Magnetic Sensor (accurate to ±4/8/12/16 gauss)
Barometric pressure	Pressure sensor (accurate to ±0.1 hectopascal)
Temperature	Temperature sensor (accurate to ±2 °C)
Humidity	Relative humidity sensor (accurate to ±4.5%)
Display	8 × 8 LED display matrix
Input	Small 5 joystick button

**Table 3:** Distribution of dataset.

Feature	Description	Normal Class		Abnormal Class	
		Mean	STD	Mean	STD
temp	Temperature	22.09583333	0.294977719	24.04901961	1.26159926
hum	Humidity	19.90416667	0.294977719	19.99019608	11.70638744
ax	The x value of accelerometer	1.557855833	3.568198363	−1.991126471	4.533231608
ay	The y value of accelerometer	2.1834275	1.362406134	4.090368627	8.006935948
az	The x value of accelerometer	15.72753	171.4913135	48.21502549	153.998157
gx	The x value of the gyroscope	−0.013850417	0.000748499	−0.012051961	0.052695636
gy	The y value of the gyroscope	−0.105782917	0.000897929	−0.01652549	0.02378498
gz	The z value of the gyroscope	0.999329167	0.003934067	0.996021569	0.053289554

**Table 4:** The significance of features presented by Information Gain (IG) Score.

Feature	IG Score
temp	1.0504
ay	0.97
gy	0.9249
hum	0.8719
gz	0.8471
gx	0.6324
az	0.4899
ax	0.4663

**Table 5:** The result of DBSCAN-based outlier detection.

# Instance (Original)	*MinPts*	*eps*	# Outlier Data	# Normal Data
342	5	7	4	338

**Table 6:** Confusion matrix of a classifier.

	Classified as “Yes”	Classified as “No”
Actual “Yes”	TP	FN
Actual “No”	FP	TN

**Table 7:** Performance metrics for the classification model.

Performance Metric	Formula
Precision	TP/(TP+FP)
Recall/Sensitivity	TP/(TP+FN)
Accuracy	(TP+TN)/(TP+TN+FP+FN)

**Table 8:** The detailed specifications of server and client computer.

		Server	Client
**Hardware**	Processor	Core i7-4790	Core i7-4790
	CPU	3.60 GHz × 8 cores	3.60 GHz × 8 cores
	RAM	16 GB	16 GB
	HDD	SSD 128 GB	SSD 128 GB
**Software**	OS	Ubuntu Server 14.04	Windows 10 Pro 64-bit
	Node.js	8.4.0	-
	Express	4.15.4	-
	Socket.IO	1.7.4	-
	Apache Kafka	0.8.2	-
	Apache Storm	0.9.3	-
	MongoDB	3.6.2	-
	JDK	-	1.8.0_121
	Eclipse	-	4.6.3
	HttpClient	-	4.5.3

**Table 9:** Performance comparison of several classification models for fault prediction.

Model	Precision (%)	Recall (%)	Accuracy (%)
Naïve Bayes (NB)	94.1	93.6	93.567
Logistics Regression (LR)	98	98	97.953
Multilayer Perceptron (MLP)	96.8	96.8	96.784
Random Forest (RF)	98.5	98.5	98.538
DBSCAN + NB	96.8	96.7	96.74
DBSCAN + LR	98.6	98.5	98.52
DBSCAN + MLP	98.8	98.8	98.81
Hybrid Prediction Model (DBSCAN + RF)	100	100	100
